# Multidimensional assessment of structural post-tuberculosis lung sequelae and respiratory health status in adults from northern Peru

**DOI:** 10.3389/fmed.2026.1889408

**Published:** 2026-07-23

**Authors:** Christian Alberto Rodriguez-Saldaña, Luis Gabriel Farfán-Chávez, Daniel Reyes-Chavez, Darwin Paul Ayala-Cespedes, Guiovanna Wong-Terrones, Mercedes del Pilar Barranzuela-Acosta, Jian Pierre Espinoza-Herreros, Ygnacio Yovani Moreno-Herrera, Gary Juan Ramos-Manchego, Elmer Cordova-Calle, Victor Serna-Alarcón

**Affiliations:** 1 Department of Infectious Diseases, Peru-Korea Santa Rosa II-2 Hospital, Piura, Peru; 2 Research and Advanced Methods for Scientific Evidence (RAMSE), Piura, Peru; 3 Human Medicine Program, Antenor Orrego Private University, Piura, Peru; 4 Department of Pneumology, José Cayetano Heredia III-1 Hospital, EsSalud, Piura, Peru; 5 Belen Hospital, Trujillo, Peru; 6 Department of Internal Medicine, José Cayetano Heredia III-1 Hospital, EsSalud, Piura, Peru; 7 School of Medicine, Cesar Vallejo University, Piura, Peru; 8 Hospital Regional de Moquegua, Moquegua, Peru

**Keywords:** dyspnea, quality of life, post-tuberculosis lung disease, spirometry, pulmonary tuberculosis, computed tomography

## Abstract

**Background:**

Tuberculosis survivors often develop persistent respiratory sequelae, but data integrating structural abnormalities, functional impairment, symptoms, and respiratory health status from Latin America remain limited.

**Aim:**

To assess structural post-tuberculosis lung sequelae and their association with respiratory health status in adults from northern Peru.

**Methods:**

We conducted a prospective analytical cross-sectional study of 106 adults previously treated for pulmonary tuberculosis and evaluated at least 6 months after treatment completion. Structural sequelae were defined as fibrosis, bronchiectasis, or residual cavitary changes on non-contrast high-resolution chest CT. Dyspnea severity was assessed with the modified Medical Research Council scale and respiratory health status with the St George’s Respiratory Questionnaire. Associations were examined using robust Poisson, ordinal logistic, and linear regression models.

**Results:**

Structural sequelae were present in 40.6% of participants, while multidimensional respiratory burden reached 59.4%. Older age was associated with structural sequelae (aPR 1.020, 95% CI 1.010–1.040), whereas longer time since treatment completion was inversely associated (aPR 0.950, 95% CI 0.920–0.980). Structural sequelae were associated with greater dyspnea severity (OR 7.680, 95% CI 3.110–20.120) and higher SGRQ scores (*β* 11.410, 95% CI 6.200–16.610).

**Conclusion:**

Structural post-tuberculosis lung sequelae were common and were associated with worse respiratory health status, supporting structured respiratory follow-up after treatment.

## 1 Introduction

Tuberculosis remains a major cause of morbidity and mortality worldwide, but its consequences extend beyond the end of antimicrobial treatment. A global modelling study estimated that 155 million tuberculosis survivors were alive in 2020, indicating that the population exposed to long-term post-treatment complications is large and clinically relevant (1). A subsequent global reappraisal of the lifetime burden of incident tuberculosis showed that post-tuberculosis sequelae accounted for 58 of 122 million disability-adjusted life years, representing nearly half of the total burden attributable to incident disease (2).

The clinical significance of this post-treatment phase is further supported by long-term outcome data. In a systematic review and meta-analysis, people treated for tuberculosis had substantially higher all-cause mortality than controls, with a pooled standardized mortality ratio of 2.91, rising to 3.76 among those classified as cured or having completed treatment (3). Respiratory impairment is also frequent after successful therapy. A recent systematic review and meta-analysis of 61 studies reported abnormal spirometry in 59.1% of treated pulmonary tuberculosis survivors, with obstructive, restrictive, and mixed abnormalities observed across settings and populations (4).

Current evidence supports a broader view of health after tuberculosis. A recent clinical statement emphasized that post-TB health includes respiratory, cardiovascular, neurological, psychological, social, and economic consequences, rather than a single organ-specific outcome (5). In parallel, a recent review in The Lancet Respiratory Medicine argued for structured prevention, diagnosis, and care of post-tuberculosis lung disease, reflecting a shift from treatment completion as the main clinical endpoint toward longitudinal assessment of post-treatment morbidity (6).

This change in perspective has also been accompanied by efforts to standardize terminology and phenotyping. Recent reviews have noted that post-tuberculosis lung disease remains under-recognized in routine practice and that differences in case definitions have limited comparability across studies (7). Earlier international consensus work also underscored the need for clearer operational definitions and harmonized research priorities in post-tuberculosis lung health (8).

The biological basis of persistent respiratory impairment after tuberculosis is well described. Chronic inflammation, matrix degradation, immune dysregulation, and abnormal tissue repair have been implicated in post-TB lung damage, providing a mechanistic basis for fibrosis, bronchial distortion, airflow limitation, and loss of lung function (9). Consistent with this, a systematic review linked previous tuberculosis with chronic respiratory disease and long-term pulmonary impairment, reinforcing the concept that microbiological cure does not imply restoration of normal lung health (10).

The consequences of tuberculosis after treatment are not confined to respiratory symptoms or imaging abnormalities. Tuberculosis survivors have a higher risk of major adverse cardiovascular events, with a pooled risk ratio of 1.51 in a recent meta-analysis (11). Socioeconomic effects also persist after treatment. In a prospective mixed-methods study from Malawi, employment and income remained lower 1 year after treatment completion than before disease onset, while extreme poverty increased significantly (12).

Despite the growing literature on post-tuberculosis morbidity, clinically detailed data from Latin America remain limited, particularly studies integrating structural abnormalities, functional impairment, dyspnea severity, and respiratory health-related quality of life within the same analytical framework. This gap is relevant in high-burden settings where post-treatment respiratory symptoms may remain unrecognized or be attributed to other conditions. The present study therefore assessed structural post-tuberculosis lung sequelae and their association with respiratory health status among adults previously treated for pulmonary tuberculosis in northern Peru.

## 2 Materials and methods

### 2.1 Study design and reporting framework

We conducted a prospective analytical cross-sectional study at Hospital III José Cayetano Heredia, a referral hospital in Piura, northern Peru, between 2021 and 2024. The manuscript was structured according to the STROBE recommendations for observational studies (13).

### 2.2 Participants

Adults aged 18 years or older were eligible if they had a previous microbiologically confirmed diagnosis of pulmonary tuberculosis established by smear microscopy, culture, or molecular testing, had completed anti-tuberculosis treatment, and were evaluated at least 6 months after treatment completion as part of a protocolized post-tuberculosis follow-up assessment. We excluded patients with chronic lung disease documented before the tuberculosis episode, including chronic obstructive pulmonary disease, asthma, or pulmonary fibrosis, those with acute respiratory infection at the time of evaluation, and those with incomplete clinical, imaging, or functional data. Consecutive eligible participants who were available, willing to participate, and provided written informed consent were included. The final sample comprised 106 participants.

### 2.3 Outcomes and variables

The primary outcome was structural post-tuberculosis lung sequelae, defined as fibrosis, bronchiectasis, or residual cavitary changes on non-contrast high-resolution chest CT performed at least 6 months after completion of anti-tuberculosis treatment. This outcome was selected as the main endpoint because post-treatment imaging abnormalities represented the most objective respiratory domain available in the final analytical framework, while the broader post-TB assessment remained multidimensional, in line with current clinical standards for PTLD evaluation (14).

To characterize respiratory impairment after tuberculosis more comprehensively, we also evaluated functional and symptomatic domains. The functional domain was defined by an abnormal spirometric pattern, and the symptomatic domain by dyspnea corresponding to a modified Medical Research Council score of at least 1. A sensitivity outcome, termed multidimensional respiratory burden, was defined as the presence of at least one abnormal domain, whether structural, functional, or symptomatic.

Dyspnea severity measured with the modified Medical Research Council scale was analyzed as an ordinal secondary outcome. The scale was selected because it provides a simple clinical measure of disability related to breathlessness and has shown meaningful associations with exercise limitation and health status in chronic respiratory disease (15). Respiratory health-related quality of life was assessed using the St George’s Respiratory Questionnaire total score and analyzed as a continuous secondary outcome. The SGRQ was chosen because it is a validated self-administered measure of health status in chronic airflow limitation and related respiratory conditions (16).

Prespecified explanatory variables were age, sex, body mass index, smoking exposure categorized as ever versus never smoking, HIV infection, number of previous tuberculosis episodes classified as one versus two or more, pulmonary cavitation documented during active disease, diabetes mellitus, and time since treatment completion expressed in months.

### 2.4 Data collection and measurements

Eligible participants were identified from hospital medical records and institutional tuberculosis databases. Demographic and clinical variables, including tuberculosis treatment history, comorbidities, active-disease cavitation, and treatment completion date, were extracted using a structured anonymized data collection form.

Spirometry, dyspnea assessment, and respiratory health status assessment were performed prospectively by the research team as part of the protocolized post-tuberculosis follow-up evaluation. Spirometry was performed by a pulmonologist according to the 2019 American Thoracic Society/European Respiratory Society technical standard, including pre- and post-bronchodilator assessment, acceptability criteria, and repeatability requirements (17). For analytical purposes, spirometric patterns were classified using predefined operational thresholds: obstruction was defined as post-bronchodilator FEV1/FVC < 0.70, a spirometric restrictive pattern as FVC < 80% predicted with FEV1/FVC ≥ 0.70, and a mixed pattern as FEV1/FVC < 0.70 with FVC < 80% predicted. Because lung volumes were not measured, the restrictive category was interpreted as a spirometric restrictive pattern rather than confirmed restriction (18). Dyspnea was assessed using the modified Medical Research Council scale, and respiratory health status was measured using the St George’s Respiratory Questionnaire.

Chest computed tomography assessment was based on non-contrast high-resolution chest CT examinations performed at least 6 months after completion of anti-tuberculosis treatment as part of the post-tuberculosis follow-up evaluation. Radiological findings were obtained from institutional CT reports issued by radiologists according to routine hospital workflow. Structural post-tuberculosis lung sequelae were defined as fibrosis, bronchiectasis, or residual cavitary changes. CT findings were extracted using a structured form and clinically corroborated by two internists from the research team. CT examinations performed during active tuberculosis were not used to define post-treatment structural sequelae.

### 2.5 Statistical analysis

Categorical variables were summarized as frequencies and percentages. Continuous variables were summarized as mean and standard deviation or median and interquartile range according to their distribution. Exact 95% confidence intervals were calculated for the main prevalence estimates.

Because the primary outcome was common, factors associated with structural post-tuberculosis lung sequelae were examined using Poisson regression with robust variance, and results were reported as adjusted prevalence ratios with 95% confidence intervals. This modeling strategy was selected because it directly estimates prevalence ratios in cross-sectional studies and avoids the inflation that may occur when odds ratios are used for frequent outcomes (38). Candidate covariates were selected *a priori* based on clinical relevance and included age, sex, body mass index, smoking exposure, HIV infection, diabetes mellitus, number of previous tuberculosis episodes, pulmonary cavitation, and months since treatment completion. Body mass index and diabetes mellitus were evaluated during model development but were not retained in the parsimonious model because they were not associated with the structural CT-based outcome, did not materially change the main estimates, and increased model complexity relative to the number of structural events. The final model included age, sex, smoking exposure, HIV infection, number of previous tuberculosis episodes, pulmonary cavitation, and months since treatment completion. Given the limited events-per-variable ratio, the adjusted multivariable model was interpreted as exploratory, and estimates were considered potentially unstable.

The association between structural post-tuberculosis lung sequelae and dyspnea severity was evaluated using ordinal logistic regression, and results were expressed as odds ratios with 95% confidence intervals. The association between structural sequelae and St George’s Respiratory Questionnaire total score was assessed using linear regression with HC3 robust standard errors. Exploratory analyses for the forced expiratory volume in one second to forced vital capacity ratio and forced vital capacity percent predicted were performed using linear regression with robust standard errors. Potential non-linearity in continuous predictors was explored using natural splines; linear terms were retained because spline-based models did not improve model fit. Multicollinearity was assessed using variance inflation factors. No formal adjustment for multiple testing was applied; therefore, findings from secondary and exploratory models, particularly borderline estimates, were interpreted cautiously. All analyses were performed in R version 4.5.0. A nominal two-sided *p* value below 0.05 was considered statistically significant.

### 2.6 Ethics

The study was approved by the Research Ethics Committee of Hospital Regional José Cayetano Heredia III-1, Piura, Peru (approval No. 000014-CEI-GR-RAPI-ESSALUD-2020; issued on November 29, 2020). All participants provided written informed consent before study participation. All data were anonymized prior to analysis to ensure confidentiality.

## 3 Results

A total of 106 adults with a history of treated pulmonary tuberculosis were included. The median age was 43.5 years (IQR, 35.0 to 54.0), 64.2% were male, and the median time since treatment completion was 15.0 months (IQR, 11.0 to 22.0). Baseline characteristics and respiratory outcome measures, overall and stratified by structural sequelae status, are presented in Table 1.

**Table 1:** Baseline characteristics and respiratory outcome measures by structural sequelae status.

**Variable**	**Overall (*n* = 106)**	**No structural sequelae (*n* = 63)**	**Structural sequelae (*n* = 43)**	***p*-value**
Demographic and clinical characteristics				
Age, years	43.5 (35.0–54.0)	41.0 (32.5–51.0)	49.0 (42.5–59.5)	0.001
Male sex	68 (64.2)	41 (65.1)	27 (62.8)	0.972
Body mass index, kg/m^2^	23.3 ± 3.7	23.0 ± 3.8	23.7 ± 3.4	0.296
Ever smoking	48 (45.3)	26 (41.3)	22 (51.2)	0.42
Diabetes mellitus	29 (27.4)	17 (27.0)	12 (27.9)	1
HIV infection	14 (13.2)	9 (14.3)	5 (11.6)	0.917
≥2 previous TB episodes	21 (19.8)	12 (19.0)	9 (20.9)	1
Pulmonary cavitation	47 (44.3)	32 (50.8)	15 (34.9)	0.156
Months since treatment completion	15.0 (11.0–22.0)	17.0 (13.0–23.0)	13.0 (10.0–17.0)	0.009
Respiratory outcome measures				
mMRC ≥1	54 (50.9)	20 (31.7)	34 (79.1)	–
SGRQ total score	21.6 ± 14.5	15.5 ± 8.9	30.6 ± 16.4	–
FEV1/FVC ratio	0.8 ± 0.1	0.8 ± 0.1	0.7 ± 0.1	–
FVC, % predicted	89.5 ± 13.0	96.0 ± 8.7	79.9 ± 12.3	–

Data are presented as n (%), mean ± standard deviation, or median (interquartile range), as appropriate. *p*-values are reported only for demographic and clinical characteristics; respiratory outcome measures are shown descriptively and were analyzed in adjusted models. Abbreviations: mMRC, modified Medical Research Council; SGRQ, St George’s Respiratory Questionnaire; FEV1, forced expiratory volume in one second; FVC, forced vital capacity; TB, tuberculosis.

Structural post-tuberculosis lung sequelae on chest computed tomography were identified in 43 participants, corresponding to a prevalence of 40.6% (95% CI, 31.1 to 50.5). When structural, functional, and symptomatic domains were considered jointly, the prevalence of multidimensional respiratory burden increased to 59.4% (95% CI, 49.5 to 68.9). Dyspnea of at least mMRC grade 1 was present in 50.9% of participants. Normal spirometry was observed in 64.2% of the sample, whereas obstructive, restrictive, and mixed spirometric patterns were found in 19.8, 10.4, and 5.7%, respectively (Table 2).

**Table 2:** Prevalence and multidimensional distribution of post-tuberculosis abnormalities.

**Variable**	**n/N**	**%**
Main prevalence estimates		
Structural post-tuberculosis lung sequelae on chest computed tomography	43/106	40.6
Multidimensional respiratory burden	63/106	59.4
Dyspnea (mMRC ≥1)	54/106	50.9
Spirometric patterns		
Normal	68/106	64.2
Obstructive	21/106	19.8
Restrictive	11/106	10.4
Mixed	6/106	5.7
Overlap between structural, functional, and symptomatic domains		
No abnormal domain	43/106	40.6
Structural + functional + symptomatic	30/106	28.3
Symptomatic only	20/106	18.9
Structural + functional	8/106	7.5
Structural + symptomatic	4/106	3.8
Structural only	1/106	0.9

Structural sequelae were defined as fibrosis, bronchiectasis, or residual cavitary changes on post-treatment non-contrast high-resolution chest CT. Functional impairment was defined by an abnormal spirometric pattern; restrictive denotes a spirometric restrictive pattern, as lung volumes were not measured. Symptomatic impairment was defined as mMRC ≥1. Multidimensional respiratory burden was defined as ≥1 abnormal domain. mMRC, modified Medical Research Council; CT, computed tomography.

**Figure 1:**
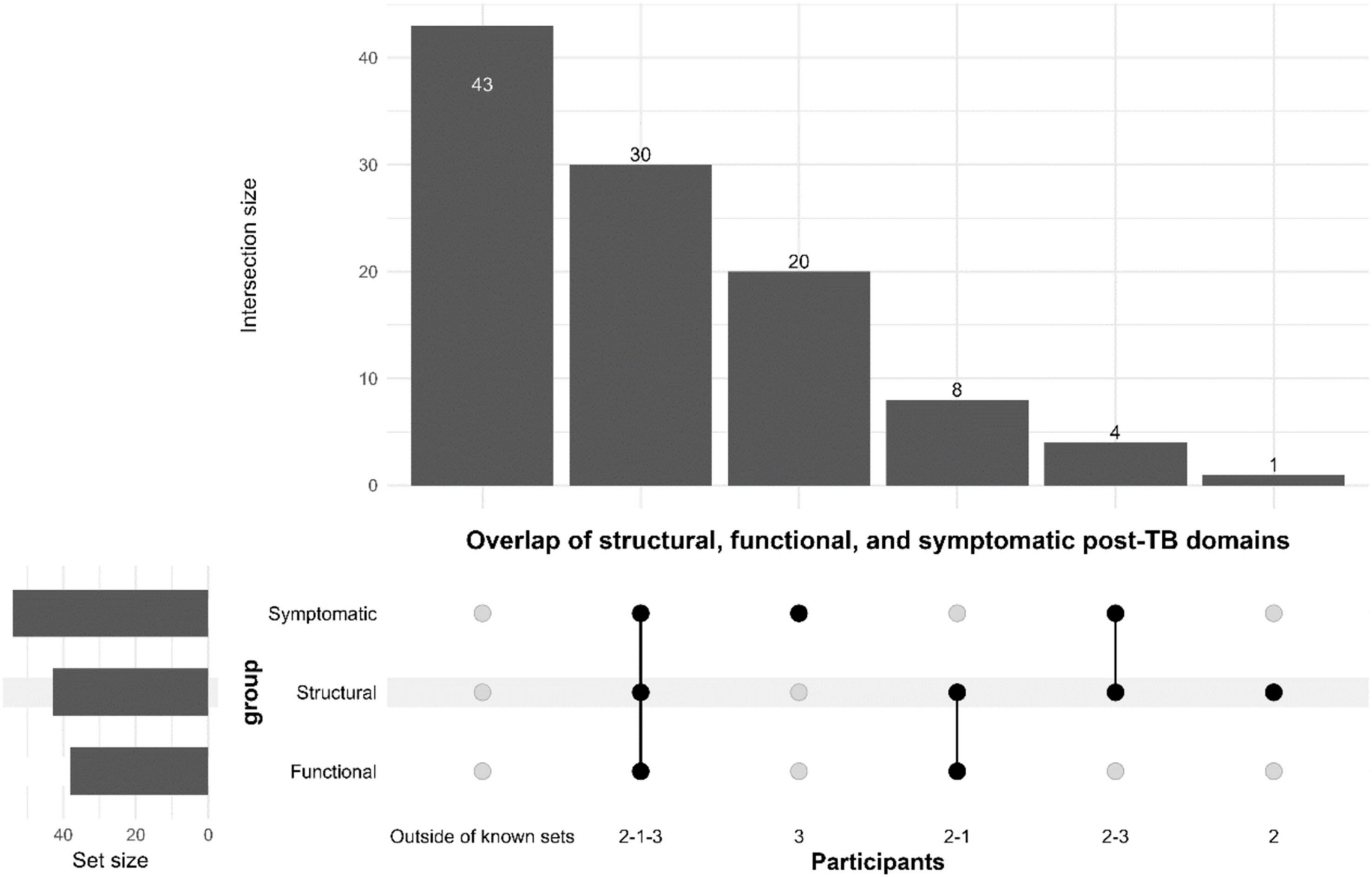
Overlap of structural, functional, and symptomatic post-tuberculosis respiratory abnormalities. UpSet plot showing the co-occurrence of structural abnormalities on chest computed tomography, functional impairment on spirometry, and symptomatic impairment defined as mMRC ≥1 among adults previously treated for pulmonary tuberculosis. Bars represent the number of participants in each intersection, and horizontal bars indicate the size of each domain. mMRC, modified Medical Research Council.

The overlap between structural, functional, and symptomatic domains is shown in Figure 1. Forty-three participants had no abnormal domain, whereas 30 had concurrent structural, functional, and symptomatic impairment. Isolated symptomatic impairment was observed in 20 participants, combined structural and functional abnormalities without symptoms in 8, structural and symptomatic abnormalities with normal spirometry in 4, and isolated structural abnormalities in 1. All participants with abnormal spirometry also had structural abnormalities on chest computed tomography (Table 2 and Figure 1).

**Figure 2:**
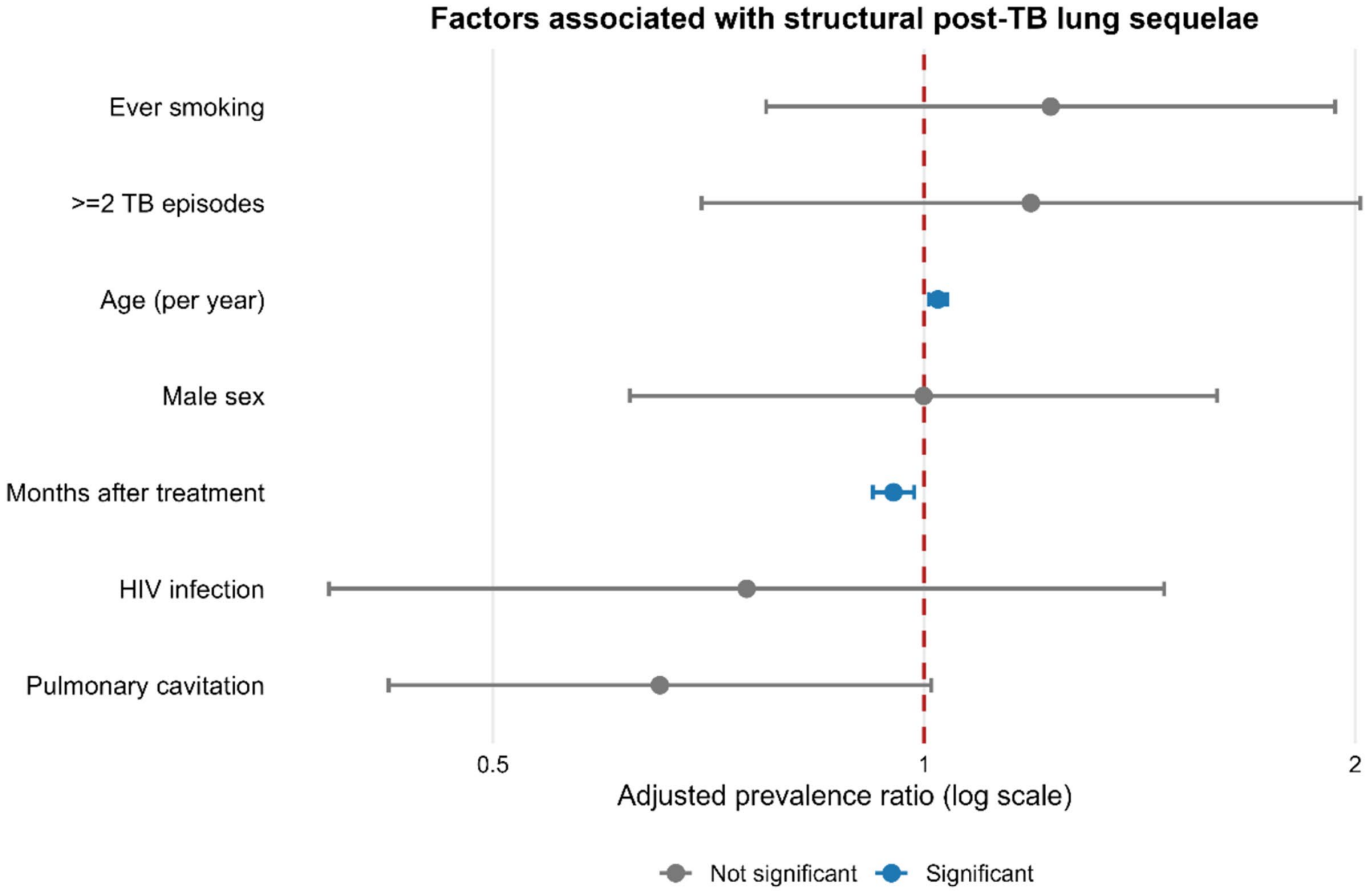
Adjusted factors associated with structural post-tuberculosis lung sequelae. Forest plot of adjusted prevalence ratios and 95% confidence intervals from the multivariable Poisson regression model evaluating factors associated with structural post-tuberculosis lung sequelae. The vertical reference line indicates an adjusted prevalence ratio of 1.0.

In the multivariable Poisson regression model, older age was independently associated with a higher prevalence of structural post-tuberculosis lung sequelae (adjusted prevalence ratio [aPR] per year, 1.020; 95% CI, 1.010 to 1.040; *p* = 0.003), whereas longer time since treatment completion was associated with a lower prevalence (aPR per month, 0.950; 95% CI, 0.920 to 0.980; *p* = 0.004). Male sex, ever smoking, HIV infection, and a history of two or more tuberculosis episodes were not associated with the primary outcome. Pulmonary cavitation showed an inverse association of borderline statistical significance (aPR, 0.650; 95% CI, 0.420 to 1.010; *p* = 0.056). Full model estimates are presented in Table 3 and graphically summarized in Figure 2.

**Table 3:** Multivariable analysis of factors associated with structural post-tuberculosis lung sequelae.

**Variable**	**Adjusted prevalence ratio** **(95% CI)**	***p*-value**
Age, per year	1.020 (1.010 to 1.040)	0.003
Male sex	1.000 (0.620 to 1.600)	0.997
Ever smoking	1.230 (0.780 to 1.940)	0.383
HIV infection	0.750 (0.380 to 1.470)	0.405
≥2 previous TB episodes	1.190 (0.700 to 2.020)	0.525
Pulmonary cavitation	0.650 (0.420 to 1.010)	0.056
Months since treatment completion	0.950 (0.920 to 0.980)	0.004

Estimates were obtained using Poisson regression with robust variance and are reported as adjusted prevalence ratios with 95% confidence intervals. Structural post-tuberculosis lung sequelae were defined as fibrosis, bronchiectasis, or residual cavitary changes on post-treatment non-contrast high-resolution chest CT. aPR, adjusted prevalence ratio; CI, confidence interval; TB, tuberculosis.

In the ordinal logistic regression analysis, structural sequelae were strongly associated with greater dyspnea severity on the mMRC scale (odds ratio [OR], 7.680; 95% CI, 3.110 to 20.120; *p* < 0.001). No other covariate showed a statistically significant association with dyspnea severity (Table 4).

**Table 4:** Association of structural post-tuberculosis lung sequelae with dyspnea severity and respiratory health-related quality of life.

Variable	Adjusted effect estimate (95% CI)	*p-*value
(A) Ordinal logistic regression for dyspnea severity (mMRC)		
Structural sequelae	7.680 (3.110 to 20.120)	<0.001
Age, per year	0.990 (0.960 to 1.020)	0.592
Male sex	1.670 (0.720 to 4.030)	0.239
Ever smoking	0.600 (0.260 to 1.360)	0.226
HIV infection	0.550 (0.150 to 1.790)	0.334
≥2 previous TB episodes	2.390 (0.900 to 6.410)	0.079
Pulmonary cavitation	1.230 (0.540 to 2.840)	0.627
Months since treatment completion	0.980 (0.930 to 1.030)	0.389
(B) Linear regression for SGRQ total score with HC3 robust standard errors		
Structural sequelae	11.410 (6.200 to 16.610)	<0.001
Age, per year	0.100 (−0.080 to 0.280)	0.278
Male sex	0.210 (−5.150 to 5.560)	0.94
Ever smoking	3.610 (−1.330 to 8.550)	0.155
HIV infection	−0.930 (−8.040 to 6.180)	0.798
≥2 previous TB episodes	1.100 (−4.920 to 7.120)	0.721
Pulmonary cavitation	−5.100 (−9.940 to −0.260)	0.042
Months since treatment completion	−0.350 (−0.610 to −0.090)	0.010

**(A)** Reports adjusted odds ratios from ordinal logistic regression for dyspnea severity measured with the modified Medical Research Council scale. **(B)** Reports beta coefficients from linear regression with HC3 robust standard errors for St George’s Respiratory Questionnaire total score. Higher SGRQ scores indicate worse respiratory health-related quality of life. mMRC, modified Medical Research Council; SGRQ, St George’s Respiratory Questionnaire; CI, confidence interval.

In the linear regression model with HC3 robust standard errors, structural sequelae were associated with higher SGRQ total scores, indicating worse respiratory health-related quality of life (*β* = 11.410; 95% CI, 6.200 to 16.610; *p* < 0.001). Longer time since treatment completion was associated with lower SGRQ scores (*β* = −0.350; 95% CI, −0.610 to −0.090; *p* = 0.010), whereas pulmonary cavitation was inversely associated with SGRQ total score (*β* = −5.100; 95% CI, −9.940 to −0.260; *p* = 0.042) (Table 4).

In the sensitivity analysis using multidimensional respiratory burden as the outcome, age remained positively associated with the outcome (aPR, 1.016; 95% CI, 1.006 to 1.027; *p* = 0.002), whereas the remaining covariates were not significantly associated (Supplementary Table S1). Diagnostic plots for the SGRQ linear model are provided in Supplementary Figure S1.

## 4 Discussion

The present study shows that structural post-tuberculosis lung sequelae remain common among adults previously treated for pulmonary tuberculosis in northern Peru and that they are associated with clinically relevant respiratory morbidity. In this cohort, structural abnormalities on chest computed tomography were identified in 40.6% of participants, while the multidimensional respiratory burden reached 59.4% when structural, functional, and symptomatic domains were considered together. Structural sequelae were also independently associated with greater dyspnea severity and worse respiratory health-related quality of life, suggesting that the radiological endpoint captured in this study may represent a clinically meaningful marker of residual morbidity rather than an incidental post-inflammatory finding.

The inverse association between time since treatment completion and structural sequelae should also be interpreted cautiously. Given the cross-sectional design and the referral-based sample, this finding should not be taken as evidence of structural recovery over time, but may reflect differences in follow-up timing, cohort composition, or selection among survivors who attended post-treatment assessment.

The magnitude of structural sequelae observed here is compatible with the broader literature, although direct comparison requires attention to outcome definition. In Tanzanian adults evaluated within 2 years of treatment completion, Mpagama et al. reported a much higher overall burden of post-TB lung disease, with persistent symptoms in 45%, abnormal spirometry in 67%, abnormal chest radiography in 86%, and a composite PTLD frequency of 91% (19). Likewise, Allwood et al. showed that substantial respiratory morbidity persists 1–5 years after TB treatment, including airflow obstruction in 38%, low FVC in 58%, and moderate-to-severe dyspnea in 25% of survivors (20). The lower prevalence in our cohort is therefore best interpreted as a function of the narrower primary endpoint, because our main analysis focused specifically on structural sequelae rather than on a broad composite definition of PTLD.

Our results also support the view that previous tuberculosis leaves durable functional consequences at both population and clinic level. In the BOLD study, a prior history of tuberculosis was independently associated with airflow obstruction and low lung function in a large multicountry population sample, with adjusted odds ratios of 2.51 for obstruction and 2.13 for low lung function (21). More detailed phenotyping from South Africa further showed that clinical patterns in PTLD are not random: obstructive disease clustered with dyspnea, chest pain, and smoking, whereas hemoptysis clustered with cavitation and fungal disease (22). This pattern is relevant to our cohort because it reinforces the idea that structural abnormalities should not be read as static radiological residues, but as part of a broader post-TB phenotype with recognizable clinical expression.

The overlap pattern in our data adds an important layer to that interpretation. Abnormal spirometry did not appear in isolation; instead, it co-occurred with structural abnormalities, suggesting that the physiological burden in this cohort was anchored to demonstrable anatomical damage. This is consistent with the Brazilian study by da Silva et al., in which lower exercise oxygen consumption was linked to reduced FVC, abnormal lung mechanics, and more severe radiographic abnormalities, including cavitation and nodular opacities (23). In that study, FVC, age, male sex, and oscillometric impairment explained a substantial proportion of VO₂peak variability, supporting the notion that structural damage after TB translates into measurable physiological limitation. Our findings extend that observation by showing that such structural damage is also strongly reflected in dyspnea severity and in patient-reported respiratory health.

The association between structural sequelae and worse dyspnea severity, together with an increase of more than 11 points in SGRQ total score, indicates that the primary outcome selected in this study is tightly connected to patient-centered morbidity. This interpretation is strengthened by the work of Pasipanodya et al., who validated the SGRQ in previously treated pulmonary tuberculosis and demonstrated excellent reliability, with an intraclass correlation coefficient of 0.927 and Cronbach’s alpha of 0.93, as well as substantially worse scores in post-TB patients than in those with latent infection (24). In practical terms, the structural lesions identified on chest computed tomography in our cohort were not neutral findings. They marked individuals with a substantially heavier symptomatic and quality-of-life burden.

This interpretation is further supported by the wider tuberculosis quality-of-life literature. Bauer et al. showed in a systematic review and meta-analysis that tuberculosis is associated with marked deterioration in health-related quality of life, with effects that remain clinically relevant beyond the acute phase (25). More specifically, Park et al. demonstrated in an EQ-5D-based systematic review that health utility improves during and after treatment, but the pre-treatment deficit is substantial, and recovery is not equivalent to full restoration of baseline health status (26). Against that background, the higher SGRQ scores observed in participants with structural sequelae in our study are biologically and clinically plausible. They suggest that radiological damage captures a residual disease state that remains visible to patients in everyday respiratory function.

Cavitation deserves a cautious interpretation. In our models, cavitation did not show a consistent positive association across outcomes, suggesting that its meaning may depend on the outcome assessed and on whether cavitation reflects active-disease severity or residual post-treatment damage. Recent evidence indicates that residual cavitary lesions may be associated with poorer post-TB quality of life, supporting their potential clinical relevance as part of a destructive structural phenotype rather than as an isolated scar (27). Post-TB lung damage may also predispose to secondary complications, including chronic pulmonary aspergillosis, particularly in patients with persistent structural abnormalities (28). However, given the number of models and coefficients evaluated and the absence of formal multiplicity adjustment, borderline cavitation estimates should be interpreted cautiously and considered hypothesis-generating.

That concern is reinforced by more recent reports specifically addressing chronic pulmonary aspergillosis in post-TB sequelae and by evidence that certain clinical and imaging characteristics are associated with hospitalization for PTLD (29, 30). Although those brief reports do not yet provide the same granularity as larger cohort studies, they support a clinically important principle: persistent structural abnormalities should not be treated as inert radiographic residues. Instead, they may define a subgroup at higher risk of recurrent symptoms, hemoptysis, fungal colonization or infection, and more intensive healthcare use. From a clinical perspective, this means that cavitary and severe structural phenotypes deserve closer follow-up than is often provided in routine TB programs.

The implications for care are immediate. If structural post-TB sequelae identify patients with worse dyspnea and poorer respiratory health status, follow-up after treatment should not end with microbiological cure. Randomized and pilot rehabilitation studies in PTLD indicate that at least part of this burden is modifiable. In Brazil, telerehabilitation improved physical capacity and quality of life after 8 weeks in patients with confirmed PTLD (31). In India, early structured pulmonary rehabilitation improved pulmonary function and six-minute walk distance, although the effect on health-related quality of life was less clear in a pilot setting (32). These studies are particularly relevant for settings like Peru, where access to formal rehabilitation may be limited and remote or simplified programs may be more scalable than conventional center-based care.

This therapeutic perspective is consistent with the broader literature arguing that PTLD should be managed as a chronic respiratory condition rather than as an epilogue to TB treatment. Singh et al. reviewed pulmonary rehabilitation in chronic post-tuberculous lung impairment and highlighted its potential to improve exercise capacity and symptoms, even when structural damage is established (33). In parallel, Visca et al. argued that post-tuberculosis sequelae require attention beyond treatment outcome and should be integrated into routine clinical follow-up and research agendas (34). The present findings give empirical support to that position because they show that imaging-defined structural sequelae remain tightly linked to outcomes that matter directly to patients.

The regional relevance of these results should also be emphasized. Qualitative evidence from Brazil shows that PTLD affects far more than respiratory symptoms, shaping stigma, daily functioning, social participation, and access to care (35). From Peru, Cabrera-Sanchez et al. reported that a previous episode of tuberculosis is associated with a higher occurrence of lung cancer, particularly in the early post-TB period, which further supports the need for long-term vigilance in survivors with prior structural lung injury (36). These findings are not directly equivalent to the outcomes measured in our study, but they widen the clinical horizon in a useful way. They show that the significance of residual post-TB damage in Latin America cannot be reduced to spirometry or chest imaging alone.

A further regional argument comes from Brazil, where national registry linkage has shown sustained excess mortality after tuberculosis, with the highest mortality rate ratios during the first years after diagnosis and persistent elevation up to 10 years later (37). Our study was not designed to evaluate survival, but the coexistence of structural damage, dyspnea, and worse respiratory health status in a Peruvian cohort is compatible with the broader evidence that the clinical consequences of tuberculosis extend far beyond treatment completion. In this sense, the present results contribute to a growing body of literature showing that survivors of pulmonary tuberculosis remain a high-risk population after cure.

Several limitations should be acknowledged. The prospective cross-sectional design precludes temporal inference and does not allow assessment of the longitudinal evolution of structural or functional abnormalities. The study was conducted at a single high-complexity referral hospital, which may have enriched the sample with more clinically complex tuberculosis survivors. Although post-treatment CT was part of a protocolized follow-up assessment rather than a symptom-triggered indication, selection bias cannot be fully excluded because participation required follow-up attendance, availability, willingness to participate, and complete respiratory assessment. Although CT findings were obtained from institutional radiology reports and clinically corroborated by the study team, formal inter-reader agreement statistics were not calculated, and complete blinding to clinical or functional information could not be guaranteed; therefore, some observer-related classification bias cannot be excluded. In addition, although CT examinations were performed at least 6 months after treatment completion, the exact interval between treatment completion and CT acquisition was not analyzed. The sample size and number of structural events limited the complexity of multivariable modeling; therefore, adjusted estimates should be interpreted as exploratory and potentially unstable. No formal adjustment for multiplicity was applied, so borderline estimates should be interpreted cautiously. Nevertheless, the study used non-contrast high-resolution chest CT as the main structural endpoint, integrated spirometry, dyspnea, and SGRQ within the same framework, and provides clinically detailed data from northern Peru, a setting still underrepresented in the PTLD literature.

## 5 Conclusion

The present study suggests that structural post-tuberculosis lung sequelae are an important marker of post-TB respiratory morbidity. These abnormalities were common and were associated with greater dyspnea severity and poorer respiratory health-related quality of life. Given the prospective cross-sectional design, the findings should be interpreted as associative rather than causal. They support structured respiratory follow-up after tuberculosis treatment, particularly among older survivors, those with persistent respiratory symptoms, and those with evidence of severe prior pulmonary injury.

## Data Availability

The raw data supporting the conclusions of this article will be made available by the authors, without undue reservation.
