# Supplementary material for: Multidimensional assessment of structural post-tuberculosis lung sequelae and respiratory health status in adults from northern Peru
Source: Front Med (Lausanne). 2026 Jul 23;13:1889408. doi: 10.3389/fmed.2026.1889408 (PMC13442519; doi:10.3389/fmed.2026.1889408)
Supplement: Supplementary file 1 [file Supplementary_file_1.docx]

Supplementary Table S1. Sensitivity analysis of factors associated with multidimensional respiratory burden after pulmonary tuberculosis

| Variable | Adjusted prevalence ratio  (95% CI) | p value |
| --- | --- | --- |
| Age, per year | 1.016 (1.006 to 1.027) | 0.002 |
| Male sex | 1.141 (0.820 to 1.587) | 0.435 |
| Ever smoking | 0.903 (0.652 to 1.252) | 0.542 |
| HIV infection | 0.769 (0.458 to 1.291) | 0.320 |
| ≥2 previous TB episodes | 1.258 (0.868 to 1.822) | 0.226 |
| Pulmonary cavitation | 0.874 (0.642 to 1.190) | 0.394 |
| Months since treatment completion | 0.981 (0.958 to 1.004) | 0.104 |

Estimates were obtained using Poisson regression with robust variance and are reported as adjusted prevalence ratios with 95% confidence intervals. Multidimensional respiratory burden was defined as the presence of at least one abnormal domain, including structural abnormalities on chest computed tomography, abnormal spirometry, or dyspnea with mMRC ≥1. Abbreviations: aPR, adjusted prevalence ratio; CI, confidence interval; mMRC, modified Medical Research Council; TB, tuberculosis.
