# Supplementary material for: Multidimensional assessment of structural post-tuberculosis lung sequelae and respiratory health status in adults from northern Peru
Source: Front Med (Lausanne). 2026 Jul 23;13:1889408. doi: 10.3389/fmed.2026.1889408 (PMC13442519; doi:10.3389/fmed.2026.1889408)
Supplement: Supplementary file 2 [file Supplementary_file_2.docx]

Supplementary Figure S1. Diagnostic plots for the linear regression model of respiratory health-related quality of life

Diagnostic plots for the linear regression model with SGRQ total score as the dependent variable, including residuals versus fitted values, normal Q-Q plot, scale-location plot, and residuals versus leverage. Higher SGRQ scores indicate worse respiratory health-related quality of life. Abbreviation: SGRQ, St George’s Respiratory Questionnaire.
